# Investigation of Hydrocolloid Plant Polysaccharides as Potential Candidates to Mimic the Functions of MUC5B in Saliva

**DOI:** 10.3390/pharmaceutics16050682

**Published:** 2024-05-18

**Authors:** Christina Winter, Carolin Tetyczka, Duy Toan Pham, Dagmar Kolb, Gerd Leitinger, Sandra Schönfelder, Olaf Kunert, Tanja Gerlza, Andreas Kungl, Franz Bucar, Eva Roblegg

**Affiliations:** 1 Institute of Pharmaceutical Sciences, Department of Pharmaceutical Technology and Biopharmacy, University of Graz, Universitätsplatz 1, 8010 Graz, Austria; christina.winter@rcpe.at (C.W.); carolin.tetyczka@rcpe.at (C.T.); 2 Research Center Pharmaceutical Engineering GmbH, Inffeldgasse 13, 8010 Graz, Austria; 3 Department of Health Sciences, College of Natural Sciences, Can Tho University, Can Tho 900000, Vietnam; pdtoan@ctu.edu.vn; 4 Core Facility Ultrastructure Analysis, Center for Medical Research, Medical University of Graz, Neue Stiftingtalstrasse 6/VI, 8010 Graz, Austria; dagmar.kolb@medunigraz.at; 5 Division of Cell Biology, Histology and Embryology, Gottfried Schatz Research Center, Medical University of Graz, Neue Stiftingtalstrasse 6/V, 8010 Graz, Austria; gerd.leitinger@medunigraz.at; 6 Institute of Pharmaceutical Sciences, Department of Pharmacognosy, University of Graz, Beethovenstraße 8, 8010 Graz, Austria; sandra.schedler@icloud.com (S.S.); franz.bucar@uni-graz.at (F.B.); 7 Institute of Pharmaceutical Sciences, Department of Pharmaceutical Chemistry, University of Graz, Schubertstraße 1, 8010 Graz, Austria; olaf.kunert@uni-graz.at (O.K.); tanja.gerlza@uni-graz.at (T.G.); andreas.kungl@uni-graz.at (A.K.)

**Keywords:** plant extracts, natural compounds, saliva, UWS, saliva substitution, xerostomia, MUC5B, in vitro adhesion, TR146 cells

## Abstract

The successful substitution of complex physiological fluids, such as human saliva, remains a major challenge in drug development. Although there are a large number of saliva substitutes on the market, their efficacy is often inadequate due to short residence time in the mouth, unpleasant mouthfeel, or insufficient protection of the teeth. Therefore, systems need to be identified that mimic the functions of saliva, in particular the salivary mucin MUC5B and the unique physiological properties of saliva. To this end, plant extracts known to contain hydrocolloid polysaccharides and to have mucus-forming properties were studied to evaluate their suitability as saliva substitutes. The aqueous plant extracts of *Calendula officinalis*, *Fucus* sp. *thalli*, and lichenan from Lichen islandicus were examined for composition using a range of techniques, including GC-MS, NMR, SEC, assessment of pH, osmolality, buffering capacity, viscoelasticity, viscoelastic interactions with human saliva, hydrocolloid network formation, and in vitro cell adhesion. For this purpose, a physiologically adapted adhesive test was developed using human buccal epithelial cells. The results show that lichenan is the most promising candidate to mimic the properties of MUC5B. By adjusting the pH, osmolality, and buffering capacity with K_2_HPO_4_, it was shown that lichenan exhibited high cell adhesion, with a maximum detachment force that was comparable to that of unstimulated whole mouth saliva.

## 1. Introduction

The salivary mucins are a family of glycoproteins that play a critical role in maintaining the essential functions of the mouth. They support the lubrication of the oral cavity and bolus formation after food intake, protecting the oral tissues and teeth from injury or invasion by xenobiotic substances [1,2,3,4]. From a chemical viewpoint, mucins can be considered as multiblock copolymers, having protein backbones and polyelectrolyte domains with flexible polysaccharides that are connected by less-glycosylated regions [5]. Since mucins have both hydrophobic and hydrophilic regions, they can form a variety of chemical interactions, such as hydrogen or disulfide bonds, as well as Van der Waals and ionic interactions [5,6]. In combination with other salivary components, such as proteins, electrolytes, and water, a complex gel-like fluid with cohesive and adhesive properties is formed [2,7]. Among the currently investigated glycoproteins, the mucin MUC5B is considered especially important, since it has been identified as the major mucin contributing to the viscoelasticity and micro-network of saliva [8,9,10]. MUC5B has a molecular weight between 2 and 40 MDa and is composed of approximately 19% protein components and 81% polysaccharides [4]. It consists of a core peptide with a well-defined amino acid sequence that is connected to a protein backbone rich in proline, threonine, and serine amino acids. This conformation enables both N- and O-glycosylation [4,10]. The most repetitive polysaccharides are N-acetyl-neuraminic acid, fucose, galactose, N-acetylglucosamine, and N-acetylgalactosamine [10,11]. MUC5B O-glycans are usually composed of five monosaccharides, which differ in composition, branching, length, and modification by sulfation or acetylation [11]. These polysaccharide side chains form the viscoelastic salivary micro-network, which remains stable even under shear stresses during swallowing or speaking [4,12,13,14]. Furthermore, the hydrocolloid polysaccharide components can effectively form hydrogen bonds, resulting in a high water-uptake capacity [6,14].

Clinical studies have shown that changes in the structure of MUC5B are closely associated with diseases [15,16,17]. For example, Chaudhury et al. reported that changes in the glycosylation patterns of MUC7 and MUC5B occur in the saliva of patients suffering from Sjögren’s syndrome [15]. Therefore, these structural changes significantly contribute to the loss of salivary functions, leading to dry mouth, which, consequently, impairs swallowing and speech and increases susceptibility to oral infections. Other clinical investigations demonstrated that radiation therapy in the head–neck area leads to the disruption of the mucin micro-network of saliva [17]. As a result, the water-retention and adhesive functions of saliva are weakened or lost, contributing to the development of xerostomia (i.e., dry mouth) and oral mucositis [18,19].

The state-of-the-art therapeutic interventions to reduce dry mouth or slow down the development of oral mucositis are topical treatments, such as saliva-replacement fluids or gels containing polysaccharides, such as carboxy-methylcellulose, glycerol, or polyethylenoxide; xanthan gum; or mucins [20,21,22]. However, several studies have shown that the effect of most marketed saliva substitutes is limited [14,19,23,24]. The disadvantages include a short duration of action, an unpleasant feeling in the mouth, or a limited effect on the prevention of enamel and dentin demineralization [14,19,25]. One explanation for this is that saliva-replacement fluids do not contain substances that carefully mimic the structure and gel-forming functions of human MUC5B. While the utilized polysaccharides are often similar to saliva regarding their viscosity, their microstructure and stability under shear stress differ significantly from salivary mucins [14,19,20,21,22]. Additionally, the currently used mucins for saliva substitutions are derived from abundant animal species such as pigs, snails, or jellyfish, but the extraction of natural products from animal tissues is difficult because of batch-to-batch variability and high risks of pathogen contamination [26,27]. Synthetic mucins have gained increased attention, but their synthesis remains a major challenge. As alternatives, hyaluronic acid [17] and natural hydrocolloids obtained from plants can be used [17,28,29]. Regarding the latter, aqueous plant extracts contain various interesting hydrocolloid polysaccharides that have so-called mucilaginous effects, a term that includes both lubricating and protective properties [30]. While some natural hydrocolloids contain high degrees of starch, which is composed of low-molecular-weight sugars, such as glucose, maltose, or maltotriose, and is hydrolyzed by α-amylase in saliva, this effect is less pronounced in patients suffering from xerostomia. Studies report not only that the lack of saliva reduces food moistening and bolus formulation but also that the damage of serous salivary cells further results in significantly less α-amylase production [31]. Consequently, polysaccharides from plant extracts could be potential candidates for saliva substitution, without being subject to hydrolyses. This applies especially to radiation-induced xerostomia, where the serous salivary cells are severely damaged. However, to what extent the microstructure and characteristics of hydrocolloids in plant extracts resemble those of human salivary mucins has not been clarified yet.

This study aimed at investigating plant extracts that contain hydrocolloid polysaccharides and are known to have mucilaginous effects. Based on the study by Schmidgall et al., *Calendula officinalis* (Calendula; flos), *Fucus* sp. thalli (Fucus; a mixture of *Fucus vesiculosus* and *Ascophyllum nodosum*), and Lichen islandicus (*Cetraria islandica*) were selected. Aqueous extracts were prepared in the case of Calendula and Fucus, whereas instead of Lichen islandicus extract, a commercially available polysaccharide, i.e., lichenan, was used [30]. The self-prepared aqueous extracts were investigated using gas chromatography–mass spectrometry (GC-MS) to evaluate trimethylsilyl (TMS) derivatives, neutral monosaccharides as alditol acetates, and the content of uronic acid. Further details on extract composition were obtained from nuclear magnetic resonance spectroscopy (1D NMR) and size-exclusion chromatography (SEC) analyses. The protein content was quantified by BCA assays. All three formulations were investigated regarding pH, osmolality, buffer capacity, and viscoelasticity. Finally, viscoelastic interactions with human unstimulated whole mouth saliva (UWS) were conducted, and the microstructure of each extract was studied with Cryo-scanning electron microscopy (Cryo-SEM). The obtained results were compared to UWS using techniques recently published by our group [13,14,17]. In order to study the adhesion of the polysaccharides in vitro under a physiological environment, human buccal epithelial cells were cultured on Aclar sheets, and tack tests were performed [32]. To take into account the conditions during swallowing, the samples were additionally stressed with shear rates up to 100 rad/s, and the force curves were recorded. All results were compared to human saliva to evaluate the suitability of the extracts as replacement fluids.

## 2. Materials Methods

### 2.1. Preparations of Aqueous Extracts/Lyophilized Powders/Aqueous Formulations

Aqueous extracts of Calendula and Fucus were prepared from commercially obtained herbs (Kottas Pharma, Vienna, Austria). For the extraction procedure, 5.0 g of each powdered herb material were stirred with 500 mL deionized water for 24 h at 24 °C (RT), following the modified protocol by Schmidgall et al. [30]. Subsequently, the solutions were centrifuged for 20 min at 2000 rpm at room temperature (RT). The supernatants were precipitated dropwise into 96% ethanol. The mixtures were cooled to 4 °C and dialyzed for 48 h using a cellulose membrane (Mw 3500 Da, Roth, Karlsruhe, Germany); after 24 h, the water was changed. Finally, after freezing the aqueous extracts in liquid nitrogen, lyophilization was conducted using Lyovac GT2 (SRK-Systemtechnik GmbH, Sömmerda, Germany) below 6 mbar over 24 h, until dry powders of the Calendula and Fucus extracts were obtained. The yield was 476 ± 56 mg (i.e., approximately 9.52%) and 682 ± 43 mg (i.e., approximately 13.64%) per run. Lichenan powder was purchased directly from VWR International (MP Biomedicals, Navi Mumbai, India).

From each powder, a 1% aqueous formulation was prepared. Briefly, 0.1 g dried powder was weighed into a glass vial before adding 9.9 g of purified water. The formulations were stirred for 15 min at 200 rpm RT.

### 2.2. Analysis of Aqueous Plant Extracts

#### 2.2.1. GC-MS Analysis for Monosaccharides after Hydrolysis

##### Qualitative Analysis of Trimethylsilyl (TMS) Derivatives

An amount of 10 mg of the freeze-dried extracts of Calendula and Fucus was mixed with 500 μL of 2 M trifluoroacetic acid (TFA) and hydrolyzed at 121 °C for 60 min [33]. The non-soluble matter was separated by centrifugation (13,000 U/min; 5 min Biofuge pico, Heraeus, Osterode, Germany). The supernatants were dried in a stream of nitrogen, and the residues were trimethylsilylated by adding 100 μL of Sigma Sil A (Sigma, St. Louis, MO, USA) [34]. As reference compounds, 10 mg of arabinose (Aldrich, St. Louis, MO, USA), fucose, mannose, rhamnose, ribose, glucose, xylose, galacturonic acid (Roth, Karlsruhe, Germany), galactose (Sigma, St. Louis, MO, USA), and glucuronic acid (Fluka, Buchs, Switzerland) were treated in the same way. The trimethylsilyl (TMS) monosaccharides were analyzed by GC-MS using a 7890A GC-System (Agilent Technologies, Santa Clara, CA, USA) coupled to a 5975C VL MSD (Agilent Technologies) on an HP5-MS column (30 m × 0.25 mm i.d., 0.25 µm film; Agilent Technologies). The injector temperature was set to 260 °C, the interface temperature to 280 °C, and the oven programmed to increase from 80 °C to 280 °C at a rate of 6 °C/min, with an additional 15 min at 280 °C. Helium was used as carrier gas at a constant flow rate of 1.2 mL/min. An amount of 1 µL of the sample was injected at a split ratio of 100:1. An electron (EI) ion source was used at 70 eV, with a source temperature of 230 °C, a quadrupole temperature of 150 °C, and a mass scanning range of 40–600 *m*/*z*.

##### Quantification of Neutral Monosaccharides as Alditol Acetates

The quantification of alditol acetates followed the method of Blakeney et al. [35], with minor modifications. A total of 10 mg of the samples and 0.5 mg of myo-inositol as internal standard were hydrolyzed with 1 mL of 2 M TFA at 121 °C for 1 h. The dried residues were dissolved in 200 μL of 1 M ammonia and reduced to alditols by 1 mL sodium borohydride in DMSO (20 g/L) at 40 °C for 90 min; 100 µL of acetic acid was used to stop the reaction. Alditoles were acetylated by 200 μL methylimidazole and 2 mL acetic acid anhydride for 20 min at RT. An amount of 10 mL of demineralized water and 1 mL of 0.1 M sulfuric acid were added. The alditol acetates were extracted with dichloromethane, the solvent removed under nitrogen, and the dried residues taken up in 1 mL dichloromethane for GC-MS analysis on a DB-225 column (30 m × 0.25 mm, i.d., 0.25 µm film; Agilent Technologies, Santa Clara, CA, USA). The injector and interface temperature was 240 °C, the oven temperature was set to 120 °C for 1 min, then from 120 °C to 230° at a rate of 4 °C/min, with an additional 15 min at 230 °C. Helium was used as a carrier gas, at a constant flow rate of 1.2 mL/min. An amount of 1 µL of the sample was injected at a split ratio of 20:1. MS conditions were identical to those mentioned above.

Quantification was performed in comparison to reference monosaccharides (arabinose, fucose, galactose, glucose, rhamnose, ribose, mannose, and xylose), which were reduced and acetylated in the same way as described before. Response factors were calculated according to the following formula:

*Rf* = *cSTD* × *AISTD*/(*cISTD* × *ASTD*) (1)

where *cSTD* is the concentration of the monosaccharide in the standard solution, *cISTD* the concentration of the internal standard in the standard solution, *AISTD* the peak area of the internal standard in the standard solution, and *ASTD* the peak area of the monosaccharide in the standard solution.

#### 2.2.2. Quantification of Uronic Acids

Uronic acids were quantified spectrophotometrically according to the method of Blumenkrantz and Asboe-Hansen [36]. In short, 5 mg of the lyophilized extracts was hydrolyzed by sulfuric acid/tetraborate, as described previously. After cooling in an ice bath, 20 µL of a solution of 0.15% 3-hydroxybiphenyl in 0.5% sodium hydroxide was added, and the absorbance was measured at 520 nm after 10 min. A calibration line was established using a 1:1 mixture of glucuronic and galacturonic acid (y = 0.4458x − 0.3465; R^2^ = 0.9959).

#### 2.2.3. Quantification of Proteins

The protein content was quantified using a Pierce™ BCA Protein Assay Kit (Thermo Fisher, Waltham, MA, USA). Samples were incubated with standards at 37 °C for 30 min and subjected to photometric measurement at 562 nm, in comparison to a BSA standard curve, according to the manufacturer’s instructions. To 25 µL of extract samples prepared as 1:10, 1:100, and 1:1000 dilutions in water or BSA standard dilutions (0–750 µg/mL), 200 µL of BCA working solution was added.

#### 2.2.4. Size-Exclusion Chromatography (SEC)

The SEC experiments were carried out on a Shimadzu UFLC system (Tokyo, Japan). A Superdex 75 PC 10/300 GL column (Cytiva, Marlborough, MA, USA) was used for separation. The flow rate of the separation buffer, composed of 136 mM sodium chloride and 10 mM sodium phosphate, was set to 0.4 mL/min. The temperature of the column oven was set to 25 °C, and the detection was conducted at 220 nm. Prior to the measurement, 10 mg of the plant extracts was diluted in a 1 mL separation buffer, mixed, and remnants were centrifuged. A total of 20 µL of the remnant free supernatant was loaded onto the column, and the running time was set to 120 min, which corresponds to 2 column volumes. A molecular weight marker from Supelco (Merck, Darmstadt, Germany), with proteins between 15 and 600 kDa (thyroglobulin 670 kDa, gamma globulin 150 kDa, ovalbumin 44.3 kDa, ribonuclease A 13.7 kDa, p-Aminobenzoic acid 0.137 kDa), was used as molecular weight reference.

#### 2.2.5. NMR Analysis

For NMR spectroscopy, the proton spectra were recorded for the extracts of Calendula and Fucus with a 700 MHz Avance II NMR spectrometer (Bruker, Rheinstetten, Germany) equipped with a cryo-probe. The solvent was D_2_O, and the experimental temperature was 25 °C.

### 2.3. Investigation of pH, Osmolality and Buffer Capacity

The aqueous, non-buffered formulations were investigated regarding pH-values (pH-meter, Lab 860, Schott Instruments, Rye Brook, NY, USA, calibrated between pH 4 and 9 at 25 °C), and the osmolality was determined by freezing-point depression (Osmomat O30-D Gonotec, Berlin, Germany). The buffer capacity was evaluated via acid titration. For this purpose, 0.01 M HCl was added dropwise to a 1 mL solution using a burette until a pH of 4.0 was reached. All samples were gently stirred at 150 rpm [14,37]. The buffer capacity of all samples was calculated using the Van Slyke formula (Formula (2)):

β = ΔCa/(ΔpH)(2)

where the buffer capacity is defined as β (mol/ĺpH, defined as slyke), ΔCa (mol/L) as the amount of acid added, and ΔpH as the change in pH achieved by the acid addition [14,35].

To adjust the buffer capacity to UWS as closely as possible, different systems were tested, i.e., potassium dihydrogen phosphate (K_2_HPO_4_), glucose, glycerol, and urea (see Supplementary Materials). Based on the data, 0.02 M K_2_HPO_4_ showed the most promising results and was thus used for further experiments.

### 2.4. Investigation of Viscoelastic Behavior

Viscoelastic characteristics, i.e., the elastic modulus (G′), the viscous modulus (G″), and the complex viscosity were determined using a Physica MC301 rheometer (Anton Paar, Graz, Austria), and the loss factor tan δ, which is the ratio of G″/G′, was calculated. As measurement system, a cone–plate geometry (CP-50-1) was used, and oscillatory measurements were performed between 0.1 and 100 rad/s at 37 °C to mimic the oral physiological conditions during swallowing. To prevent liquid evaporation during the measurement, a built-in evaporation hood was used. The results were compared to prior investigations carried out with UWS [13,14].

### 2.5. Investigation of Viscoelastic Interactions with Human Saliva

Five ml of UWS was collected from each of three healthy volunteers (two female and one male), as described in previous studies [14,17]. The fresh saliva samples were centrifuged (30 min at 2000 rpm) at 4 °C to remove cell and food debris, as recommended by Zhang et al., and the supernatant was used for all further steps [38].

A 1:1 mixture of UWS supernatant and each plant extract was prepared to evaluate the viscoelastic interactions. The mixture was gently stirred at 50 rpm for 2 min, which corresponds to the maximum residence time of mouthwashes in patients with dry mouth [39]. Next, the viscoelastic properties were determined at 37 °C with the same test setup as described in Section 2.4. and used to calculate interaction parameters, as described by Rossi et al. [40,41]. Briefly, the viscoelastic parameters were calculated as differential values to assess Δη/η, ΔG′/G′ and ΔG″/G″ using Formulas (2)–(5).

Δη/η_mixture_, where Δη = η_mixture_ × (η_plant extract_ + η_UWS_) (3)

ΔG′/G′_mixture_, where ΔG′ = G′_mixture_ − G′_plant extract_ (4)

ΔG″/G″_mixture_, where ΔG″ = G″_mixture_ − G″_plant extract_ (5)

To consider the effects of physiological shear strain in the oral cavity, the value at the lowest angular frequency (0.1 s^−1^) and that at the highest angular frequency (100 s^−1^) were considered for the calculation.

### 2.6. Investigation of Microstructure

To investigate the microstructure of the hydrocolloidal polysaccharides present in the aqueous formulations, the Cryo-SEM technique (Quorum PP3010T, Quorum Technologies, Laughton, East Sussex, UK) was employed. Prior to visualization, the samples were frozen under slush liquid nitrogen and transferred with a vacuum transfer device into the preparation chamber, for subsequent processing and observation. The preparation chamber was connected to a GEMINI Sigma 500 (ZEISS Company, Oberkochen, Germany) SEM, including a nitrogen gas cold stage. For observation, the samples were fractured, sublimated, and sputter-coated with palladium in the chamber. The material was transferred into the SEM specimen chamber before image recording. Images were taken using a 5 kV acceleration voltage, with a backscattered secondary electron detector, at magnifications between 200 and 50,000×.

### 2.7. Culturing of Human Buccal TR 146 Cells on Aclar Sheets and UWS Collection

Human buccal TR146 cells were purchased from Imperial Cancer Research Technology (London, UK) and were cultivated in Dulbecco’s Modified Eagles’s medium (DMEM, Gibco, Life Technologies Corporation, Painsley, UK), supplemented with 10% fetal bovine serum (FBS; Gibco), 1% penicillin streptomycin (Penstrep; Gibco), and 1% NEAA (MEM Non-Essential Amino Acid Solution, 100×; Sigma-Aldrich, Munich, Germany). During cultivation, the cells were kept at a constant temperature of 37 °C, in a humidified atmosphere, with 5% CO_2_. The medium was changed every second or third day and sub-cultivation of confluent cells was performed on a weekly basis using 0.25% trypsin-ethylenediaminetetraacetic acid (trypsin-EDTA; Gibco). For the mucoadhesion studies, TR146 cells were seeded on Aclar fluoropolymer sheets (ScienceServies, Munich, Germany), cut to a diameter of 26 mm to fit the PP25 measurement system, with a seeding density of 1 × 10^5^ cells/sheet, and incubated until they reached confluence [42].

### 2.8. In Vitro Adhesion Studies on TR146 Cells

**Figure 1:**
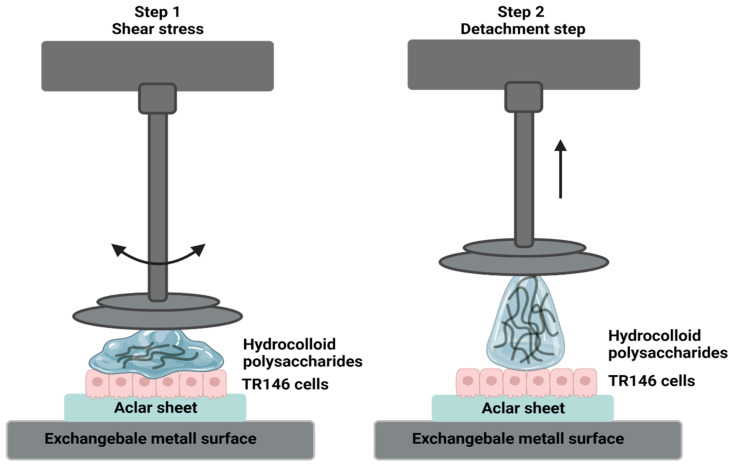
Schematic illustration of the applied in vitro adhesion setup. In step 1, shear stresses of 0.1 to 100 rad/s are applied to simulate physiological shear rates in the oral cavity. In step 2, the system detaches the aqueous solution containing hydrocolloid polysaccharides from the TR146 cell layer.

Prior to the adhesion studies, the TR146 cells were washed with a phosphate-buffered saline (PBS, pH 7.4; Gibco). The remaining liquid was removed by carefully draining it from the Aclar film. Since complete removal could not be guaranteed without endangering the cells, an Aclar film was used as a control for all measurements, which was pre-equilibrated in a cell culture medium/PBS and cleaned accordingly. Tack tests were performed using a Physica MC301 rheometer (Anton Paar, Graz, Austria) with a plate–plate geometry (PP25), at 37 °C. The Aclar sheet with the cell layer was glued to an exchangeable metal surface. To adjust the experimental setup to the natural shear stress in the oral cavity during swallowing, stresses up to 100 rad/s were applied for 30 s on the unbuffered plant formulations, as well as on UWS. Next, the stainless-steel probe was separated from the cell surface at a constant speed of 500 µm/s. During the total separation time, 500 data points were used to evaluate F_N_ and F_max_. As described in the literature, F_max_ was converted and expressed to a positive value [17,42,43]. A schematic representation of the in vitro adhesion tack tests is demonstrated in Figure 1.

### 2.9. Statistical Analysis

If not stated otherwise, the data presented originate from triple-fold determinations. The results are presented as mean values ± standard deviations (SDs). To evaluate statistical significance, the Student’s *t*-test was used, and differences were evaluated as significant at a level of *p* < 0.05 (*). If not marked otherwise, the significance is in reference to the UWS from healthy volunteers.

## 3. Results

### 3.1. Analysis of the Extract Components

Using reference compounds subjected to the same hydrolysis conditions to form anomers corresponding to the hydrolyzed extract sample, arabinose, fucose, mannose, rhamnose, ribose, glucose, galactose, xylose, galacturonic acid, and glucuronic acid were detected in Calendula. In comparison, only fucose, glucose, glucuronic acid, rhamnose, mannose, galactose, and xylose were found in Fucus (Supplementary Materials, Figures S1 and S2 and Table S4). However, quantification was not possible due to numerous overlapping peaks, which resulted from the formation of the TMS derivatives of the tautomeric forms of the monosaccharides [44]. Hence, the alditol acetates of the neutral monosaccharides were prepared. The contents of the neutral monosaccharides in the extracts are presented in Table 1. The alditol acetates could be well separated on a DB-225 column, confirming the presence of the monosaccharides in both extracts.

**Figure 2:**
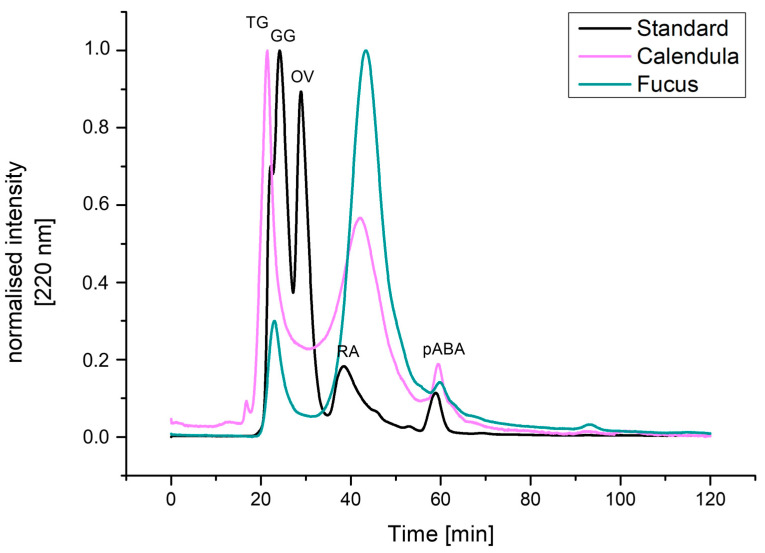
SEC of Calendula and Fucus extracts. A protein-based molecular weight marker was used as size reference. Detection was performed at 220 nm, and the intensity was normalized. TG: thyroglobulin 670 kDa, GG: gamma globulin 150 kDa, OV: ovalbumin 44.3 kDa, RA: ribonuclease A 13.7 kDa, pABA: p-Aminobenzoic acid 0.137 kDa.

The total amounts of uronic acids were determined by the 3-hydroxybiphenyl reaction with uronic acids, according to Blumenkrantz and Asboe-Hansen [36]. It was found that the extract from Calendula contains twice as much uronic acid as the Fucus extract (i.e., 556.3 ± 45.7 mg/g and 210.6 ± 32.6 mg/g, respectively). The content of BCA-positive compounds was found to be higher in Fucus compared to the Calendula extract (298.154 and 60.205 mg/g, respectively), indicating a lower protein content in the latter. However, it cannot be excluded that other compounds present in the extracts reacted with BCA as well. The analysis of the molecular weight distribution within the extracts of Calendula and Fucus was determined by SEC, in comparison to a protein-based molecular weight marker as size reference. For the Calendula extract, the molecular weight of the most prominent peaks could be backcalculated as 265,759.6 Da and 4155.2 Da, respectively, whereas the Fucus extract showed a prominent peak, with 3720.8 Da. Details are presented in Table 2 and Figure 2.

Proton NMR spectra suggest the presence of polysaccharides as the main components of the extracts, while compounds with longer alkyl chains, such as fatty acids are minor components. Protein resonances are not observed in the D_2_O samples. The ^1^H NMR spectra of both compounds are presented in Figures S3 and S4 (Supplementary Materials). The ^1^H NMR spectrum of the commercial lichenan is in accordance with the expected glucan structure (Supplementary Materials Figure S5).

### 3.2. pH, Osmolality, and Buffer Capacity

An overview of the data is summarized in Table 3. The results show that the measured pH values for the aqueous non-buffered formulations of Calendula and Fucus are acidic (i.e., 4.26 ± 0.08 and 4.62 ± 0.06), while the pH for lichenan is in the neutral range (i.e., 6.74 ± 0.09). The osmolality for all three formulations is very low. For Calendula, no value could be determined with the applied method, so it is assumed that the osmolality is close to zero. For both Fucus and lichenan, the osmolality values are 0.007 ± 0.004 and 0.001 ± 0.001 osmol/kg, respectively. The addition of K_2_HPO_4_ as a buffer system resulted in an increase in the pH values for all three extracts. Thereby, the pH values of Calendula and Fucus range from 7.02 ± 0.09 to 7.41 ± 0.09, and the pH of lichenan increased to 8.91 ± 0.08. Due to the salt-based buffer system, the osmolality of Fucus and lichenan also increased to values close to those of natural saliva, i.e., 0.053 ± 0.013 osmol/kg and 0.052 ± 0.006 osmol/kg versus 0.050 ± 0.013 osmol/kg. For Calendula, it was still not possible to determine the osmolality via freezing-point depression. The calculated buffer capacity for all three extracts is similar or even higher than that of natural saliva (i.e., 7.90 ± 0.082 for Calendula, 8.41 ± 0.31 for Fucus, 5.50 ± 0.22 for lichenan, versus 5.34 ± 1.7 mmolH^+^/L for UWS).

### 3.3. Viscoelastic Behavior and Viscoelastic Interactions with UWS

**Figure 3:**
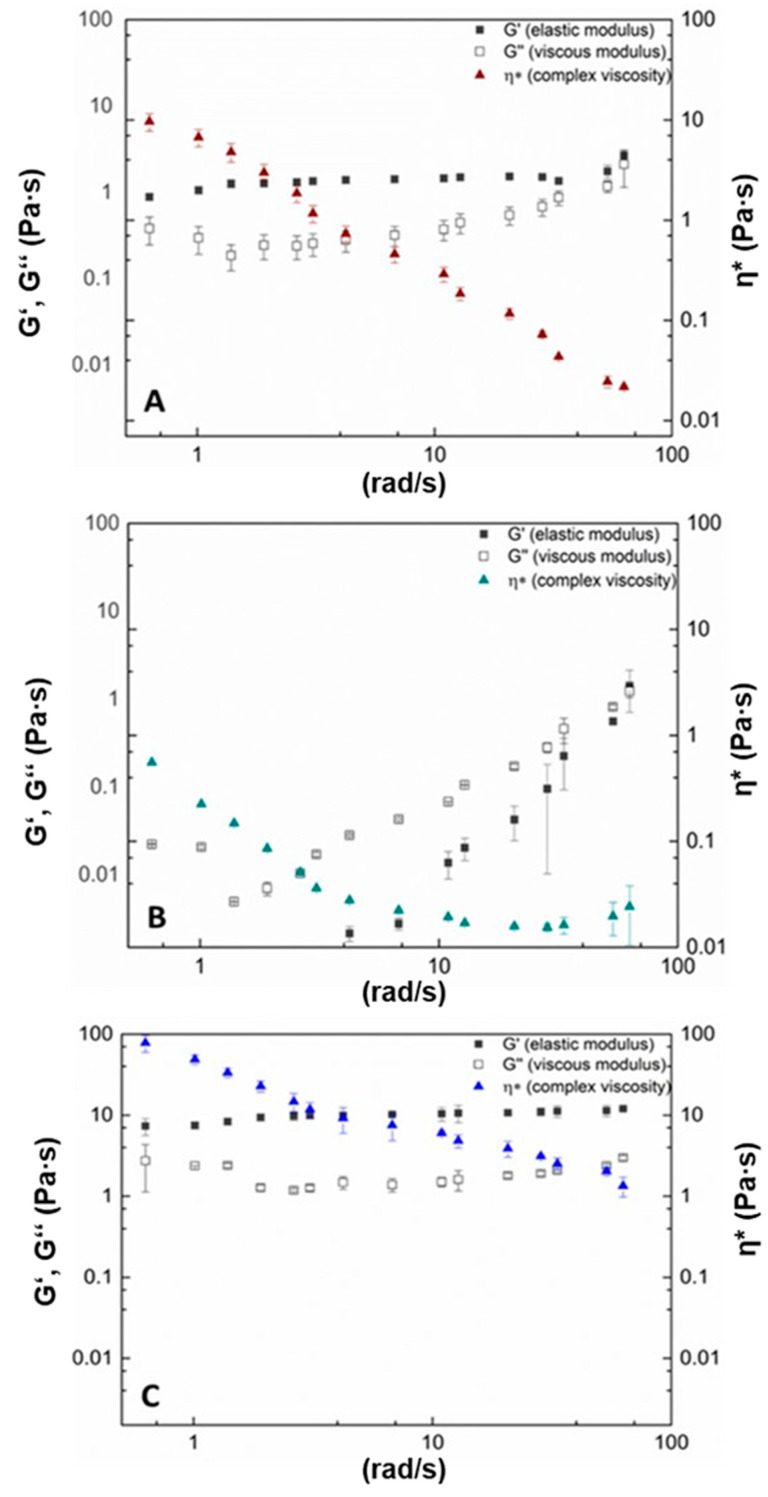
Viscoelastic behavior of 1% (*w*/*w* %) aqueous solution of Calendula (**A**), Fucus (**B**), and lichenan (**C**).

The oscillation measurements show that for both Calendula and lichenan, the elastic modulus (G′) dominates the viscous modulus (G″) over the applied shear stress (Figure 3A,C). However, for Calendula, the loss factor tan δ increases with increasing shear stress from 0.43 ± 0.04 to almost 0.79 ± 0.16. This indicates that the intramolecular network structure became weaker due to shear stress. In contrast, the tan δ for lichenan remained in a constant range, i.e., from 0.37 ± 0.09 to 0.24 ± 0.08, which suggests that the polysaccharide network structure remained unchanged during shear stress. While both Calendula and lichenan are shear-thinning fluids, the viscosity of lichenan remained about 10-fold higher than that of Calendula over the applied shear rate. Fucus, however, displayed a different viscoelastic behavior, with a very low starting viscosity of about 0.6 mPa·s and no detectable elastic portion at lower shear rates (Figure 3B). With increasing shear stress, the viscosity increased slightly, as did the elastic fraction. The tan δ for all measurement points is >1, indicating no stable crosslinked network structure.

**Figure 4:**
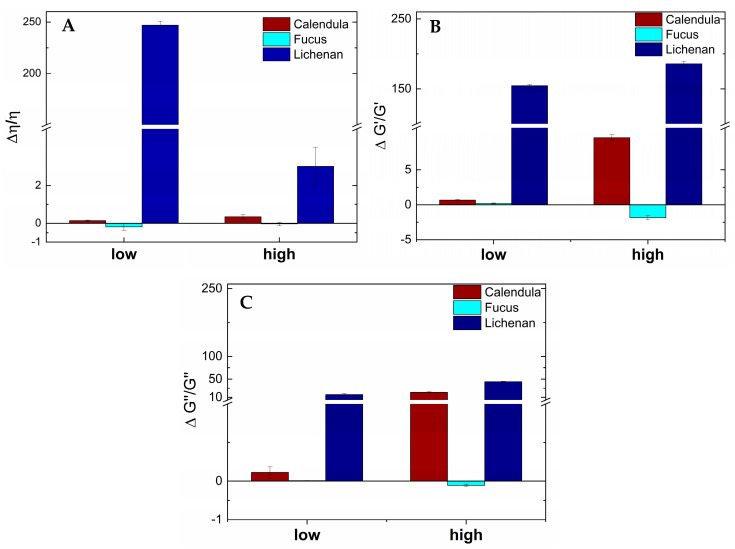
Viscoelastic parameters of viscosity (**A**), elastic (**B**) and viscous moduli (**C**) of Calendula, Fucus, and lichenan, mixed with human saliva at low (0.1 rad/s) and high (100 rad/s) shear rates.

The viscoelastic parameters were calculated from the viscosity values, G′ and G″, of the three plant extracts mixed with UWS, compared to the parameters of the plant extracts alone, as described in detail by Rossi et al. [40,41,45]. Briefly, values above zero indicate chain entanglements and non-covalent bonds between mucin chains (i.e., in our case, mostly MUC 5B from UWS [8,17]) and polymer chains (i.e., in our case, polysaccharide chains from the plant extracts) [46]. Values below or around zero signalize that no detectable interactions have occurred. The results presented in Figure 4A–C show the highest positive values for the interactions between UWS and lichenan for all three viscoelastic parameters (i.e., viscosity, elastic modulus G′, and viscous modulus G″). Interestingly, while the interactions regarding viscosity are, as expected, shear-dependent, no significant changes were detected for the interactions between G′ and G″ across low and high shear rates. For Fucus, no positive values were found for any viscoelastic parameter when mixed with UWS. The viscoelastic interactions for Calendula and UWS show diverse results. While only slightly positive values are obtained for interactions calculated from viscosity at both low and high shear rates, as well as for G′ and G″ at low shear rates, the interactions calculated from both viscoelastic moduli increase noticeably with increasing shear rates.

### 3.4. Microstructure of Hydrocolloidal Polysaccharides

**Figure 5:**
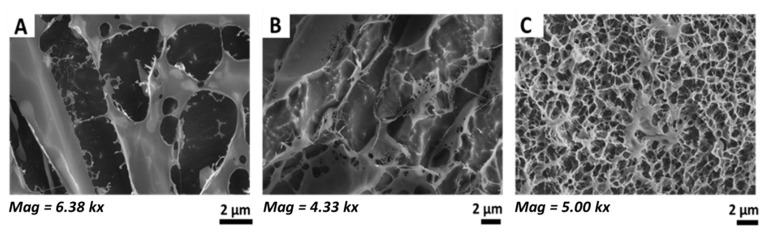
Representative Cryo-SEM images of the polysaccharide structure of Calendula (**A**), Fucus (**B**), and lichenan (**C**).

The images obtained via Cryo-SEM show fiber structures for all three investigated plant formulations; however, their arrangement and thickness vary considerably (Figure 5). The polysaccharide structure of Calendula (Figure 5A) consists of thick fibers of more than 2 µm width. These thick fibers are connected by only a few thin fibers, resulting in intermediate spaces of up to 10 µm. The polysaccharide structure of Fucus shows similar separated fibers, with many broken sections and intermediate spaces between 5 to 10 µm visible (Figure 5B). In contrast, Cryo-SEM images obtained for lichenan reveal a coherent, crosslinked network consisting of fibers and pores ranging from the nano- to low micrometer scale (Figure 5C).

### 3.5. In Vitro Adhesion on TR146 Cells

**Figure 6:**
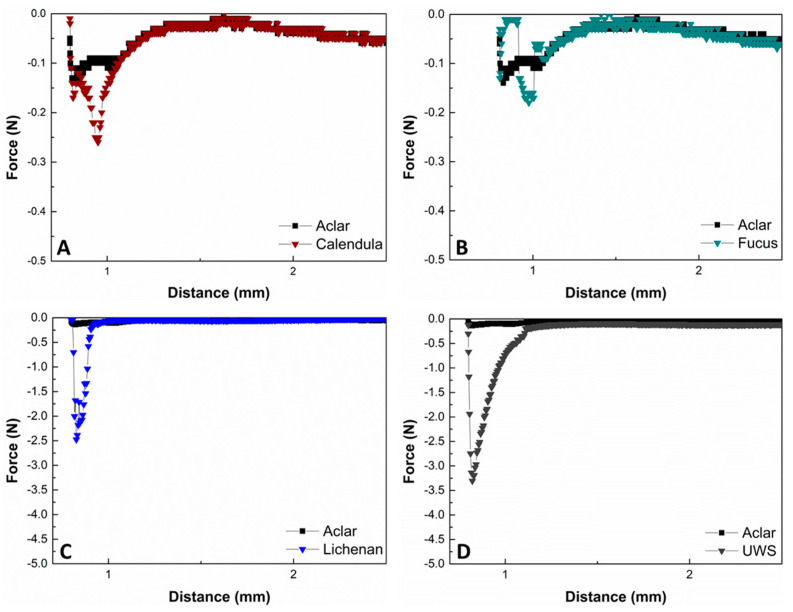
Representative force distance curves of Calendula (**A**), Fucus (**B**), lichenan (**C**), and UWS (**D**), obtained via tack tests from a coherent TR146 cell surface, compared to a blank Aclar sheet. The sheet was placed in cell medium for the same amount of time and washed with PBS.

The obtained force curves from the tack tests conducted on human buccal epithelial TR 146 cells gave an indication about the adhesive behavior of the buffered aqueous Calendula, Fucus, and lichenan formulations, compared to UWS. The maximal force F_max_ before the disruption point for both Calendula and Fucus is in a low range (i.e., 0.26 ± 0.09 N and 0.17 ± 0.04 N), while for Calendula, a small but sharp detachment curve is visible. F_max_ for Fucus is only slightly higher than the control (Figure 6A,B). For lichenan, the F_max_ is 2.09 ± 0.20 N, which is about 10-fold increased compared to that for the other two plant candidates. The adhesive performance of lichenan is closest to that of UWS, with a F_max_ of 3.30 ± 0.47 N (Figure 6C,D). UWS also displays the broadest force curve, with the disruption process completed after a distance of 1.25 ± 0.09 mm, while the distances for Calendula, Fucus, and lichenan remain below 1.00 mm.

## 4. Discussion

The successful substitution of complex physiological fluids such as human saliva has remained a challenge in pharmaceutical formulation design up to the present day. While there are a variety of saliva substitutes available on the market, their efficacy in treating dry mouth or preventing the development of, e.g., oral mucositis during and after radiation therapy, is insufficient or only of short duration [14,19,47,48,49,50]. The reasons for their limited success is that they do not resemble human saliva in either structure or functionality [14,19,23,48]. A possible starting point is the identification of systems that recapitulate MUC5B, with additional consideration given to the physiological properties of saliva. These include pH, osmolality, and buffering capacity, as well as viscoelasticity, which involves the formation of a hydrocolloid network that remains stable under shear stress during swallowing or speaking, and adhesion to the oral mucosa [4,14,17,19,20,39]. Among others, hydrocolloid polysaccharides and other ingredients from specific plant extracts, such as *Calendula officinalis* flowers, *Fucus* sp. thalli, and Lichen islandicus, are known to provide mucilaginous effects [36] and could thus show potential to be used as salivary replacement fluids.

Calendula flowers consist, aside from flavonoids, terpenoid glycosides/esters, and carotenoids, of carbohydrates. The water-soluble polysaccharides include 25.77% acidic sugar, 31.25% reducing sugars, and 84.58% pectic substances, as well as various mono-saccharides, including glucose, arabinose, rhamnose, xylose, galactose, and galacturonic acid. Varljen et al. isolated three homogenous polysaccharides with a (1→3)-linked β-D-galactan backbone from Calendula flowers and revealed immunostimulating effects [51]. In contrast to the studies of Slavov et al. [52] and Schmidgall et al. [30], in the present study, fucose could be identified in the polysaccharide fraction of Calendula flowers. The aqueous extract contained only a low proportion of BCA-positive compounds (ca. 6%). Fucoidans consist of fucose and sulfate, while laminarans, which are algal glucans, comprise small linear polysaccharides (20–50 linked glucose residues). This matches with the major peak after the SEC of the Fucus extract, representing compounds with a molecular weight of about 4000 Da. Moreover, monosaccharides such as mannose, galactose, glucose, xylose, etc., and a protein content of 30% have been identified. Lichen islandicus consists of 25–50% polysaccharides, mainly mucilages such as lichenan and isolichenan, and 7% proteins [30]. Lichenan is a mixed β-1,3/1,4-glucan, for which effects on cellular differentiation in human keratinocytes could be shown [53]. While the specific compositions of water-soluble polymers of all three extracts differ from MUC5B, which consists mainly of N-acetyl-neuraminic acid, fucose, galactose, N-acetylglucosamine, and N-acetylgalactosamine, the mixture of long-chain polysaccharides and short-chain sugars is similar [11] Nevertheless, the micro-networks formed in aqueous solution vary in functionality and stability to different degrees. Accordingly, the extract compositions resulted in divergent physicochemical properties.

The physicochemical investigations of the prepared aqueous extracts showed that both the pH value and the osmolality of Calendula and Fucus were significantly lower than those of UWS. The acidic pH is most likely due to the presence of high amounts of uronic acids, respectively, 556.3 ± 45.7 mg/g in Calendula and 210.6 ± 32.6 mg/g in Fucus [30]. In the case of lichenan, the pH value was in the neutral range, which is consistent with the data in the literature [30]. On the one hand, Lichen islandicus contains lower amounts of uronic acids; on the other hand, commercial lichenan was used instead of the whole plant. The low osmolality for all three extracts indicates that they do not contain sufficient electrolytes to be comparable to UWS, which has a neutral pH, an osmolality of 0.052 ± 0.003 osmol/kg, and a buffer capacity higher than 5.00 mmol H^+^/L [7,14,50]. However, a neutral pH range and a high buffer capacity are essential to oral health, as these characteristics prevent the demineralization of the teeth. In principle, it is relatively easy to adjust the pH and buffer capacity by adding a simple salt-based buffer system, but it is a challenge to remain in the low hypotonic osmolality range. This is crucial, as the hypotonicity of natural saliva enables normal taste function and the expansion and hydration of mucin side chains [54,55]. Additionally, excessive osmolality during saliva substitution leads to a loss of water from the oral epithelial cells, which would be counterproductive for patients suffering from xerostomia, when the product is taken for prolonged periods [14,53]. The buffer system of natural UWS is very complex and multifaceted, consisting of bicarbonates, phosphates, urea, histidine-rich peptides, and others [54,55]. Preliminary investigations showed that, while the buffer solutions of salt-based buffers, glucose, glycerol, and urea alone could be adjusted to the desired characteristics (i.e., an osmolality between 0.5 and 0.6 osmol/kg with neutral or low basic pH), the aqueous plant extracts were not compatible with most of the investigated buffer systems (see Supplementary Materials). Only by adding the salt-based buffer (0.02 M K_2_PO_4_) could the required osmolality, as well as the same or even higher buffer capacity than UWS, be achieved. For lichenan, the resulting pH was slightly in the basic range, which could cause dental plague if used frequently as a saliva substitute without further modification [14,19]. This aspect is to be considered in future studies.

For sufficient lubrication, hydration, and adhesion, as well as a pleasant mouthfeel, viscosity and viscoelastic properties are especially important. The viscoelasticity and the micro-network of UWS are among the most crucial characteristics of UWS in a healthy state. It is well described in the literature that UWS is a shear-thinning fluid, with the elastic portion larger than the viscous one during shear stresses caused by swallowing and speaking [4,7,13,48,54]. These characteristics result from the salivary microstructure, which consists of a coherent network formed mainly by MUC5B fibers with a high water-uptake capacity. While the mechanisms of mucoadhesion are still not completely understood and are usually described as a combination of several theories (i.e., electronic theory, wetting theory, adsorption theory, diffusion theory, mechanical theory, and fracture theory), the hydrated, gel-like network of UWS seems to be one of the main driving forces for adhesion to the oral mucosa [17,55,56,57,58,59,60]. The rheological investigations in this study showed that, regarding viscoelasticity and network formation, lichenan acts similarly to UWS. As the elastic modulus dominates the viscous one over the entire range of applied shear stress, the network remains stable during physiological shear stresses. Furthermore, the polysaccharide chains of lichenan showed strong viscoelastic interactions with mucins from UWS. While the interactions were highest at low shear rates, the chain entanglements and non-covalent interactions between the polysaccharides and mucins from UWS (i.e., mostly MUC5B) remained intact, even at the highest tested shear rates that can occur in the mouth during swallowing or speaking. The Cryo-SEM images confirmed that the micro-network of the lichenan structure is dense and coherent. This is reflected in the lowest tan δ value of 0.24 ± 0.08, which was three times lower than that of UWS (i.e., 0.89), indicating the high stability of the network. Consequently, the viscosity of lichenan was also higher, which in turn might lead to an unpleasant mouthfeel if the formulation is not modified further. For both Calendula and Fucus, the viscoelasticity and the structure of the micro-network differed from that of UWS. While for Calendula, the elastic modulus also dominates the elastic one, the network seems to become less stable at higher shear rates, which is indicated by the tan δ value close to 1 (i.e., 0.79 ± 0.16 when the SD is taken into account). Additionally, for Calendula, there were positive viscoelastic interactions with UWS detected when taking both the elastic and viscous modulus into consideration. However, there were no relevant interactions when only viscosity was considered. Interestingly, the interactions increased at high shear rates, indicating that with longer interaction time, as well as additional oscillatory mixing, more covalent and non-covalent bonds were formed. For clinical application, this could mean that longer gargling times in the mouth could enable good miscibility with the remaining saliva in the oral cavity, but also with the residual salivary pellicle, thus improving adhesion to the mucosa. For Fucus, either no elastic component was detectable, or it was significantly lower than the viscous one. Although the Cryo-SEM images for Fucus suggest a network structure, the rheological investigations imply that this network is unstable under physiological shear stress. Moreover, Fucus did not show any interactions with mucins from UWS, indicating that neither covalent nor non-covalent interactions were formed to any notable extent. This essentially leads to poor adhesion to the oral mucosa and rapid swallowing of the formulation, which makes Fucus the least ideal candidate for a saliva substitute, among the extracts examined. Our observations are in accordance with other studies on mucoadhesion, where polysaccharides showed higher adhesive interactions with mucins when the initial viscosity was high [61]. This was also the case for lichenan, compared to Calendula and Fucus, which had a 10-fold higher viscosity than Calendula and an almost 100-fold higher viscosity than Fucus over the different shear rates. Additional studies report that the pH value influences the adhesive behavior [58,59,60]. For example, an aqueous whey-protein solution shows maximum interaction with mucins at a pH of 6.8 [61], while higher adhesion is observed with chitosan at an acidic pH of 5.2, which is related to the solubility and charge of the polymer in this case [62]. Our results show that the lichenan extract, which has the highest initial pH value, displays stronger mucoadhesion compared to Calendula and Fucus, which have an acidic pH value. While optimizing the pH value of the extracts might enhance the interactions with mucins, the range for fine-tuning the pH in saliva substitutes for patients suffering from radiation-induced xerostomia is limited, as it is recommended to stay close to a neutral pH [2,7,12,14].

The conclusions drawn from the rheological and microstructure investigations were cross-checked with adhesion studies. For this purpose, in vitro tack tests were performed to carefully evaluate the adhesion of the liquid extract formulations to buccal epithelial cells after applying shear stress. In order to compare the obtained data, the same experiment was performed with UWS obtained from healthy volunteers. The UWS showed the broadest detachment curve and highest F_max_ (i.e., 3.30 ± 0.47 N) from all investigated samples. Although the tan δ of UWS is significantly higher, and the viscosity is lower than that of lichenan, surprisingly, the in vitro adhesion to human buccal TR 146 cells was still improved, suggesting that the adhesive and cohesive forces of natural UWS are more pronounced. However, as expected, from all the investigated extracts, lichenan showed the highest adhesion, with a maximum detachment force F_max_ of 2.09 ± 0.20 N. In contrast, Calendula and Fucus adhered only slightly to the cells, and the values for F_max_ were almost 10-fold lower than those of UWS. The poor adhesion for Calendula and Fucus is in contradiction to the ex vivo investigations by Schmidgall et. al., who reported that a 1% extract of Calendula adhered best to porcine buccal mucosa, followed by Fucus [30]. It must be noted here that in this ex vivo study, excised porcine buccal membranes were used, which were incubated with the plant solutions in a horizontal manner, with only “gentle stirring”; thus, physiological effects were not reflected in the setup [30]. This is specifically true for physiological shear stress, which can be higher than 60 rad/s during swallowing or speaking, equaling more than 570 rpm on a magnetic stirring system [13,14]. Moreover, the pH value and buffer capacity were not considered. Currently, there is a great interest in both in vitro and ex vivo methods that allow studying mucoadhesive interactions and the delivery of drugs across both intact and diseased oral barriers [6,59,60,61,62,63]. While several approaches have been reported, including rheological tests, ellipsometry, investigation of the wetting angle on mucin films or fixed cell layers, tensile stress tests, tack tests, AFM-spectroscopy, or the falling liquid film method, each method still has its limitations and should not be used as a stand-alone method [17,36,59,64,65,66,67,68,69,70,71]. However, the presented in vitro tack test performed on a confluent cell layer under the consideration of oral shear stress provided in this study, can be a helpful approach in this regard.

## 5. Conclusions

The complex glycan lichenan from Lichen islandicus was identified as a promising candidate for mimicking the properties of MUC5B in saliva. In order to adjust the pH, osmolality, and buffer capacity to salivary conditions, K_2_HPO_4_ proved to be the most suitable, although not perfect, buffer system. The formulation matched human saliva in terms of viscoelasticity, microstructure, and network stability, even under shear stress. By developing a physiologically adapted tack test setup using a confluent cell layer and by taking into account shear stress during swallowing, the data showed that lichenan exhibited the highest adhesion, with a maximum detachment force, which was in the same range as UWS. This was further confirmed by the viscoelastic interaction studies, showing interactions between the polysaccharides and mucins from UWS, even at high shear rates. However, it should be noted that this formulation needs to be further adapted, especially with regard to mouthfeel and pH, to fulfill the requirements of an improved saliva substitute.

## Figures and Tables

**Table 1:** Monosaccharides in the Calendula and Fucus extracts after hydrolysis, quantified as alditol acetates.

	Monosaccharides [mg/g]							
Extract	Ara (#)	Fuc	Gal	Glu	Man	Rha	Rib	Xyl
Calendula	18.9	5.3	61.1	59.4	5.1	29.5	0.5	4.2
Fucus	n.d. (##)	111.7	18.5	165.0	43.0	1.0	n.d.	15.7

(#) Ara: arabinose, Fuc: fucose, Gal: galactose, Glu: glucose, Man: mannose, Rha: rhamnose, Rib: ribose, Xyl: xylose; (##) n.d. not detected.

**Table 2:** Backcalculation of the molecular weight of the different main peaks of the Calendula and Fucus extracts.

	Protein	MW [Da]	Retention Time [min]	Retention Volume [mL]	log MW
Calendula	Peak 1	265,759.6	21.1	8.4	5.4
	Peak 2	4155.2	41.2	16.5	3.6
	Peak 3	179.2	56.4	22.6	2.3
Fucus	Peak 1	225,192.0	21.9	8.8	5.4
	Peak 2	3720.8	41.7	16.7	3.6
	Peak 3	96.3	59.4	23.8	2.0

**Table 3:** Overview of pH, osmolality, and buffer capacity of the investigated extracts with and without the addition of a buffer system, as well as UWS without any additions [14].

Plant Extract	Without K_2_HPO_4_		With 0.02 M K_2_HPO_4_		
	pH	Osmolality [osmol/kg]	pH	Osmolality [osmol/kg]	Buffer Capacity [mmol H^+^/L]
Calendula	4.26 * ± 0.08	- (#)	7.02 ± 0.09	- (#)	7.90 * ± 0.82
Fucus	4.62 * ± 0.06	0.007 * ± 0.004	7.41 ± 0.09	0.053 ± 0.006	8.41 * ± 0.31
Lichenan	6.74 ± 0.09	0.001 * ± 0.001	8.91 * ± 0.08	0.052 ± 0.006	5.50 ± 0.22
UWS [14]	6.80 ± 0.17	0.052 ± 0.003	-	-	5.34 ± 1.70 (##)

(#) Not possible to determine via freezing-point depression. (##) Measured without the addition of K_2_HPO_4_. * Indicates significant differences from natural UWS.

## Data Availability

Data are contained within the article and Supplementary Materials.

## Supplementary Materials

The following supporting information can be downloaded at: https://www.mdpi.com/article/10.3390/pharmaceutics16050682/s1, Table S1: Overview of osmolality and pH of the investigated buffer systems in aqueous solution; Table S2: Overview of osmolality and pH of the plant extracts in the buffer systems to reach 1 % (*w*/*w*) extract concentration; Table S3: Response factors and relative retention of monosaccharide alditol acetates; Table S4: Retention times of TMS monosaccharide peaks used for identification of individual monosaccharides in hydrolyzed Calendula and Fucus polysaccharides (HP5-MS column); Figure S1: GC-MS total ion chromatogram of Calendula extract after TMS derivatization; Figure S2: GC-MS total ion chromatogram of Fucus extract after TMS derivatization; Figure S3: Proton spectrum (700 MHz, D_2_O) of the Calendula extract; Figure S4: Proton spectrum (700 MHz, D_2_O) of the Fucus extract; Figure S5: Proton spectrum (400 MHz, D_2_O) of lichenan.
