# Supplementary material for: Investigation of Hydrocolloid Plant Polysaccharides as Potential Candidates to Mimic the Functions of MUC5B in Saliva
Source: Pharmaceutics. 2024 May 18;16(5):682. doi: 10.3390/pharmaceutics16050682 (PMC11124828; doi:10.3390/pharmaceutics16050682)
Supplement: Supplementary file 1 [file pharmaceutics-16-00682-s001.zip › pharmaceutics-2916318-supplementary.pdf]

Supplementary Materials

S1. Evaluation of buffer system

In order to adjust the osmolality to UWS (i.e., 0.052 ± 0.003 osmol/kg), different buffer concentrations were either added to Milli-Q water and dissolved under gentle stirring at 150 rpm at RT until a clear solution was received (K_2HPO_4, Urea) or diluted accordingly with purified water (Glucose, Glycerol). The results of the most suited concentrations are summarized in Supplementary Table S1.

Supplementary Table S1 Overview of osmolality and pH of the investigated buffer systems in aqueous solution.

Buffer system	Osmolality [osmol/kg]	pH
**0.02 M K_2HPO_4**	0.054 ± 0.002	9.16 ± 0.10
**0.055 M Glucose**	0.054 ± 0.001	6.33 ± 0.09
**0.07 M Glycerol**	0.056 ± 0.002	8.48 ± 0.14
**0.06 M Urea**	0.061 ± 0.003	6.79 ± 0.07

To investigate changes in osmolality and pH, 1 % (w/w) dried extract powder was added to the prepared buffer solutions (see Supplementary Table S2).

Supplementary Table S2. Overview of osmolality and pH of the plant extracts in the buffer systems to reach 1 % (w/w) extract concentration.

Buffer system	Calendula		Fucus		Lichenan	
	**pH**	**osmolality [osmol/kg]**	**pH**	**osmolality [osmol/kg]**	**pH**	**osmolality [osmol/kg]**
**0.02 M K_2HPO_4**	7.02 ± 0.09	- (#)	7.41 ± 0.09	0.053 ± 0.006	8.91 ± 0.08	0.052 ± 0.006
**0.055 M Glucose**	4.24 ± 0.06	- (#)	5.05 ± 0.04	0.062 ± 0.004	5.9 ± 0.05	0.058 ± 0.001
**0.07 M Glycerol**	4.24 ± 0.12	- (#)	5.04 ± 0.02	0.063 ± 0.002	6.78 ± 0.03	0.060 ± 0.005
**0.06 M Urea**	4.65 ± 0.06	- (#)	5.01 ± 0.05	0.066 ± 0.007	6.78 ± 0.04	0.066 ± 0.003

(#) Not possible to determine via freezing point depression.

Supplementary Table S3. Response factors and relative retention of monosaccharide alditol acetates.

Monosaccharide	Response factor	Relative retention time(#)
Arabinose	1.093	0.761
Fucose	1.364	0.714
Galactose	1.120	0.953
Glucose	1.015	0.978
Mannose	1.127	0.930
Rhamnose	1.459	0.704
Ribose	1.021	0.750
Xylose	1.143	0.806
myo-Inositol	1.000	1.000

(#) Compared to Rt of myo-inositol on DB-225 column

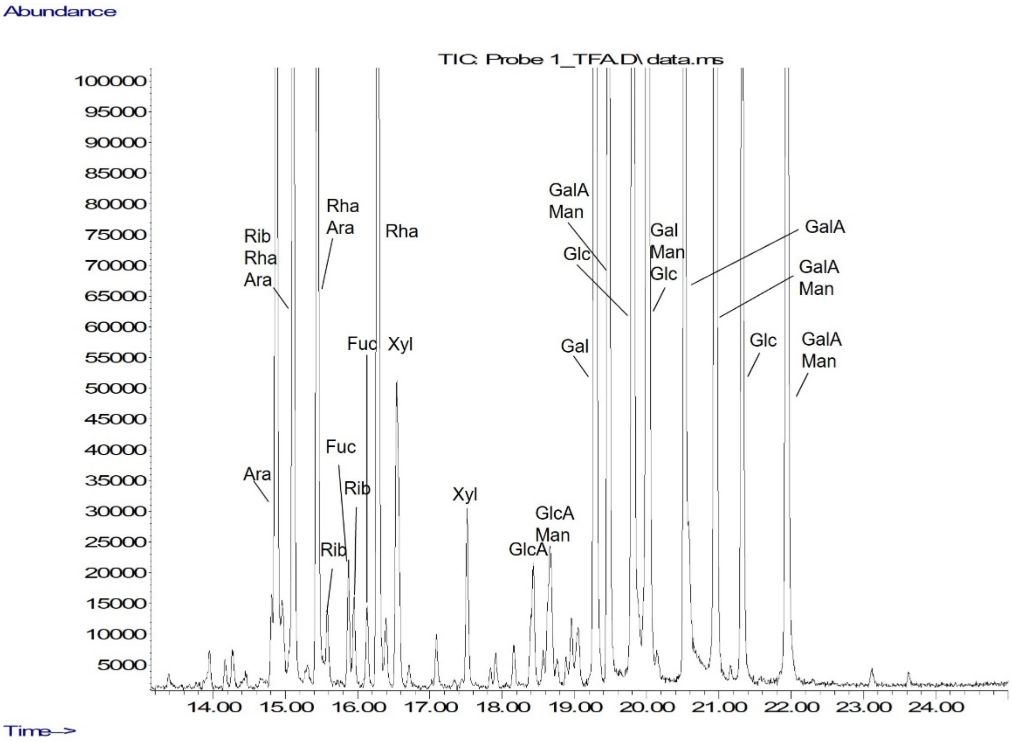

Supplementary Figure S1. GC-MS total ion chromatogram of Calendula extract after TMS derivatization. Overlapping peaks resulted from formation of TMS derivatives of tautomeric forms of the monosaccharides. Ara Arabinose, Fuc Fucose, Gal Galactose, GalA Galacturonic acid, Glc Glucose, GlcA Glucuronic acid, Man Mannose, Rha Rhamnose, Rib Ribose, Xyl Xylose.

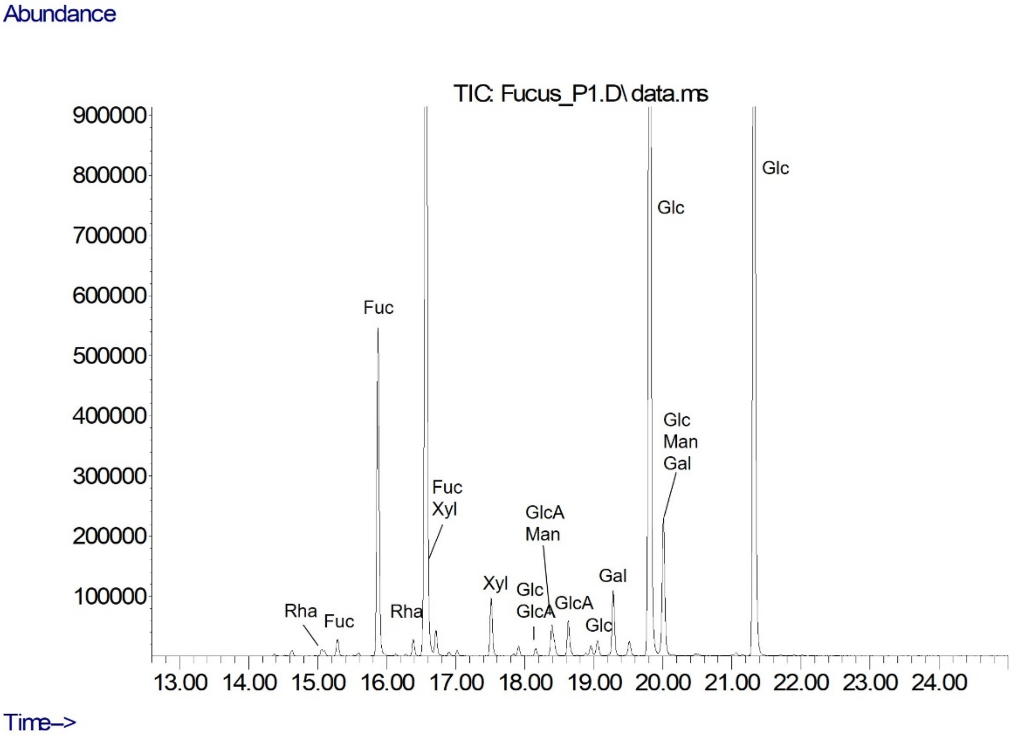

Supplementary Figure S2. GC-MS total ion chromatogram of Fucus extract after TMS derivatization. Overlapping peaks resulted from formation of TMS derivatives of tautomeric forms of the monosaccharides. Fuc Fucose, Gal Galactose, Glc Glucose, GlcA Glucuronic acid, Man Mannose, Rha Rhamnose, Xyl Xylose.

Supplementary Table S4. Retention times of TMS monosaccharide peaks used for identification of individual monosaccharides in hydrolyzed Calendula and Fucus polysaccharides (HP5-MS column).

**Compound**	**Retentions times [min]**
Arabinose	14.87, 15.27, 15.44
Fucose	15.29, 15.88, 16.09, 16.57, 17.10
Galactose	19.29, 20.02
Galacturonic acid	19.47, 20.52, 20.95, 21.94
Glucose	19.81, 19.97, 21.32
Glucuronic acid	18.04, 18.44, 18.65
Mannose	18.39, 20.00, 20.95, 21.93
Rhamnose	15.11, 15.35, 16.28
Ribose	15.02, 15.50, 15.73
Xylose	15.07, 16.54, 17.51

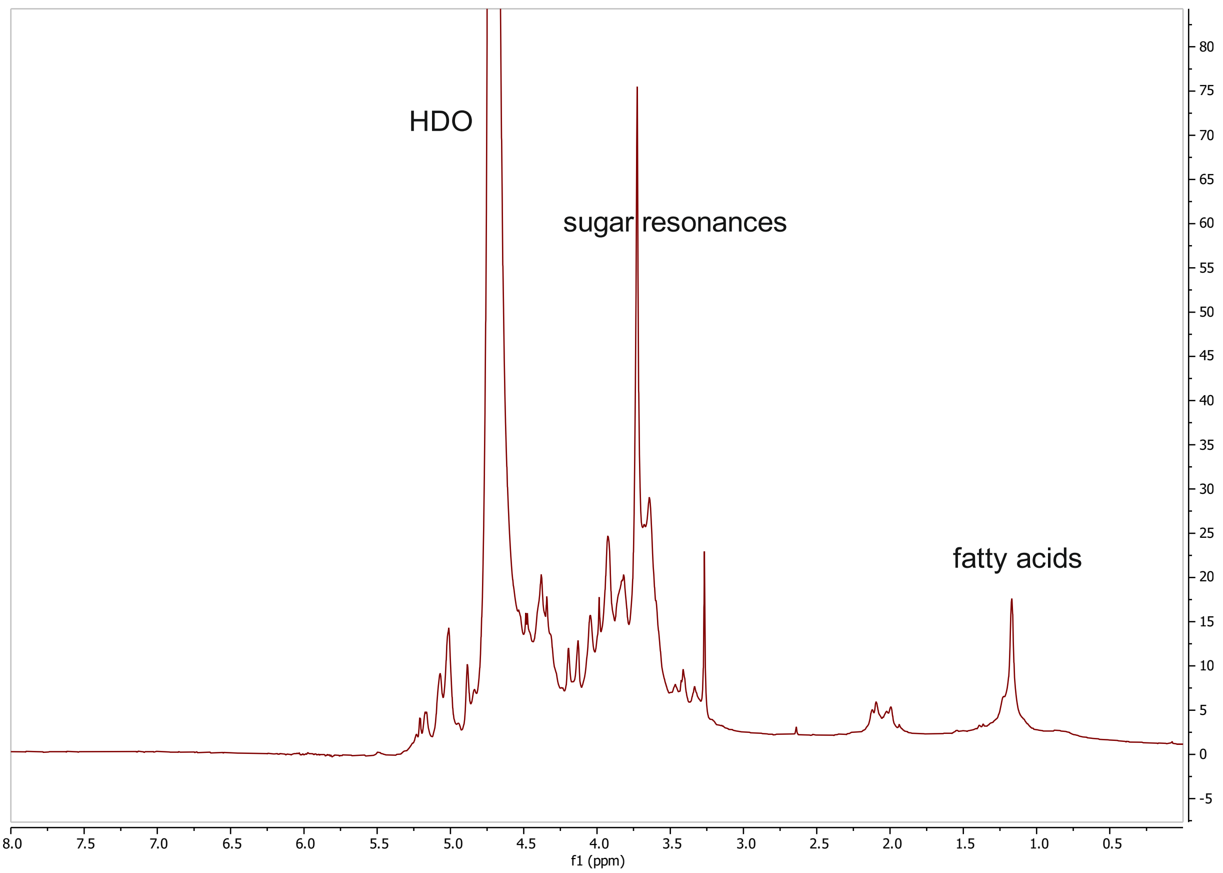

Supplementary Figure S3. Proton spectrum (700 MHz, D_2O) of the Calendula extract.

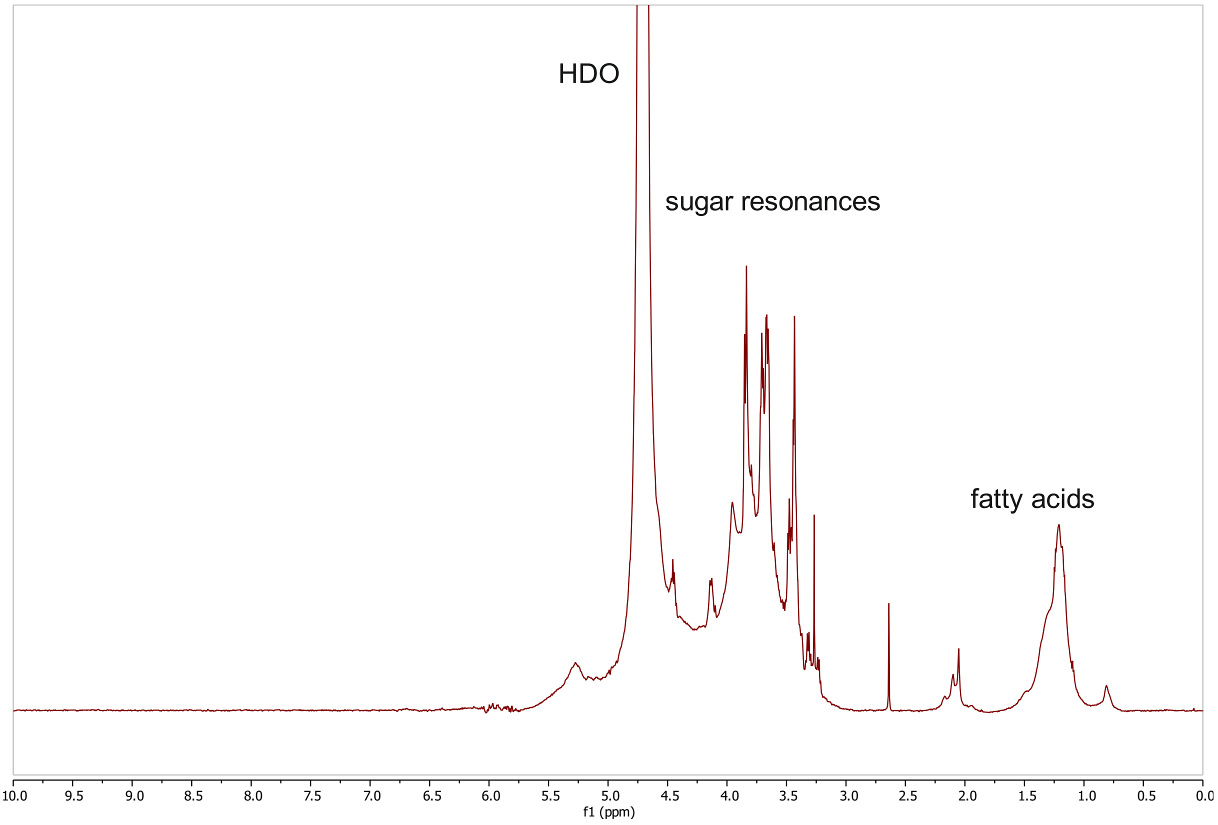

Supplementary Figure S4. Proton spectrum (700 MHz, D_2O) of the Fucus extract.

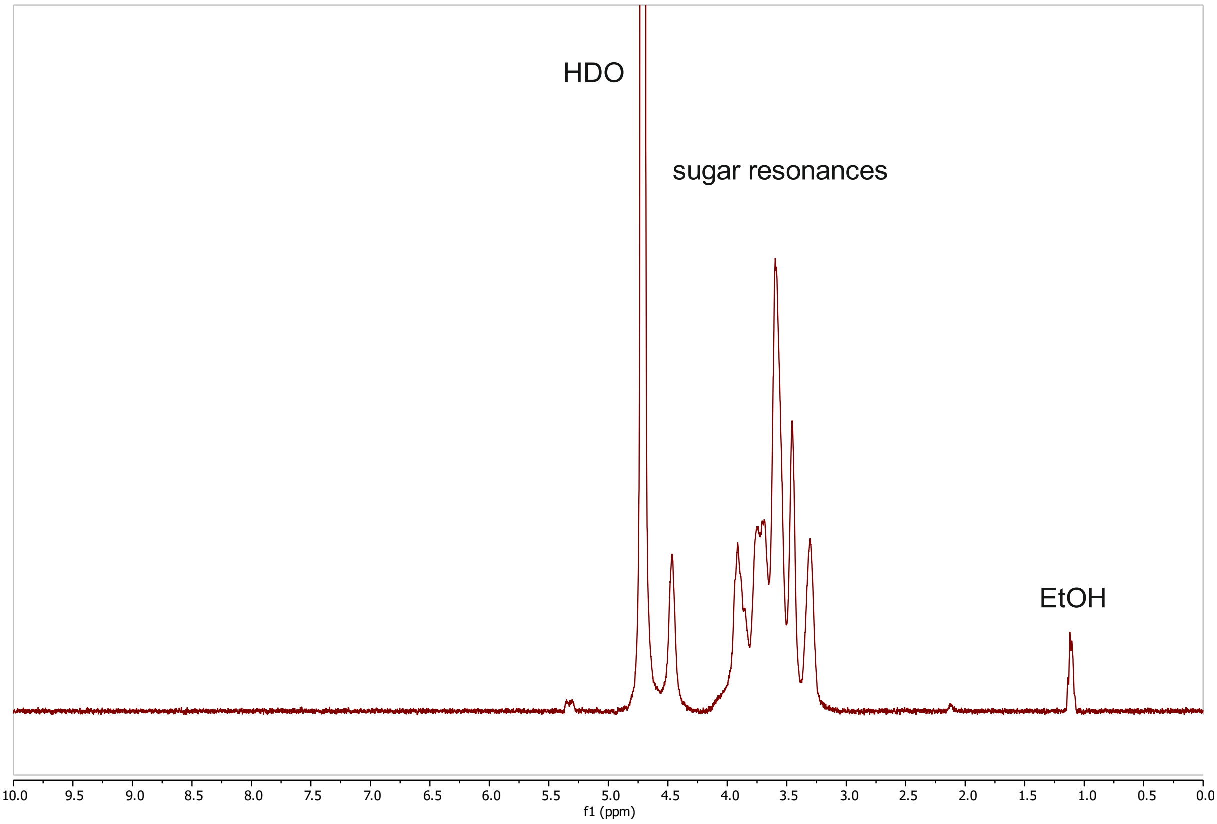

Supplementary Figure S5. Proton spectrum (400 MHz, D_2O) of lichenan.
